# Patient Trust in Physicians Matters—Understanding the Role of a Mobile Patient Education System and Patient-Physician Communication in Improving Patient Adherence Behavior: Field Study

**DOI:** 10.2196/42941

**Published:** 2022-12-20

**Authors:** Dezhi Wu, Paul Benjamin Lowry, Dongsong Zhang, Youyou Tao

**Affiliations:** 1 Department of Integrated Information Technology University of South Carolina Columbia, SC United States; 2 Department of Business Information Technology Virginia Tech Blacksburg, VA United States; 3 Department of Business Information Systems & Operations Management The University of North Carolina at Charlotte Charlotte, NC United States; 4 Department of Information Systems and Business Analytics Loyola Marymount University Los Angeles, CA United States

**Keywords:** mobile health, mHealth, trust, patient adherence, mobile patient education system, MPES, patient-physician communication, theory of planned behavior, TPB, patient-centered care, mobile phone

## Abstract

**Background:**

The ultimate goal of any prescribed medical therapy is to achieve desired outcomes of patient care. However, patient nonadherence has long been a major problem detrimental to patient health, and thus is a concern for all health care providers. Moreover, nonadherence is extremely costly for global medical systems because of unnecessary complications and expenses. Traditional patient education programs often serve as an intervention tool to increase patients’ self-care awareness, disease knowledge, and motivation to change patient behaviors for better adherence. Patient trust in physicians, patient-physician relationships, and quality of communication have also been identified as critical factors influencing patient adherence. However, little is known about how mobile patient education technologies help foster patient adherence.

**Objective:**

This study aimed to empirically investigate whether and how a mobile patient education system (MPES) juxtaposed with patient trust can increase patient adherence to prescribed medical therapies.

**Methods:**

This study was conducted based on a field survey of 125 patients in multiple states in the United States who have used an innovative mobile health care system for their health care education and information seeking. Partial least squares techniques were used to analyze the collected data.

**Results:**

The results revealed that patient-physician communication and the use of an MPES significantly increase patients’ trust in their physicians. Furthermore, patient trust has a prominent effect on patient attitude toward treatment adherence, which in turn influences patients’ behavioral intention and actual adherence behavior. Based on the theory of planned behavior, the results also indicated that behavioral intention, response efficacy, and self-efficacy positively influenced patients’ actual treatment adherence behavior, whereas descriptive norms and subjective norms do not play a role in this process.

**Conclusions:**

Our study is one of the first that examines the relationship between patients who actively use an MPES and their trust in their physicians. This study contributes to this context by enriching the trust literature, addressing the call to identify key patient-centered technology determinants of trust, advancing the understanding of patient adherence mechanisms, adding a new explanation of the influence of education mechanisms delivered via mobile devices on patient adherence, and confirming that the theory of planned behavior holds in this patient adherence context.

## Introduction

### Background

The primary goal of any prescribed medical therapy is to achieve the desired medical outcomes of patient care, which requires a certain level of patient adherence, a critical measure of quality care. Previous studies have shown that nonadherence results in an economic burden of approximately US $100 to US $300 billion per year in the United States [1] owing to the costs of disease progression, readmissions, wasted resources, labor burden, and insurance costs [2], representing 3% to 10% of total health care costs in the United States [3,4]. Another meta-analysis of 79 studies across several countries showed that all-cause nonadherence costs ranged from US $5271 to US $52,341 per person [5]. In a medical context, adherence is defined as patients’ behaviors that coincide with health care providers’ health and medical advice, such as taking prescribed medication and following suggested diets [6-8]. For optimal therapeutic efficacy, adherence rates of >80% are needed, and for patients with certain more serious conditions, adherence rates of >95% are required [7,9]. Many health intervention programs have been implemented in different health care settings, but low success rates have persisted, imposing a major financial burden on the US health care system [5,10]. Studies have shown that the average adherence rate for long-term medication therapies is 40% to 50%, but the adherence rate for short-term therapies is 70% to 80% [11-13]. Even with clinical performance incentives and tremendous efforts regarding the development and examination of interventions, the current rate of long-term adherence to evidence-based medications in cardiometabolic diseases remains low [10]. Patients who comply with a treatment, even when the treatment is a placebo, have better health outcomes than those with poor adherence [14]. In contrast, patients who do not comply with the recommended treatments cause unsuccessful medical interventions and therapies, which exacerbate undesired health outcomes such as suboptimal therapeutic outcomes, delayed recovery, and more additive or aggressive treatments with the potential for more adverse events [4,15]. Thus, therapeutic adherence has been a long-standing topic of clinical concern for decades owing to the widespread nature of nonadherence.

Poor patient adherence can obscure a clinician’s assessment of therapeutic effectiveness and result in avoidable hospitalization, increased mortality risk, and increased health care costs [5,8,16]. Furthermore, because of undetected or unreported therapeutic nonadherence, physicians may change the regimen, which may increase the cost or complexity of a treatment, thus further increasing the burden on patients. According to the Centers for Medicare and Medicaid Services, national prescription drug spending is expected to continue growing by approximately 6% per year from 2023 to 2028 [17]. Such nonadherence is extremely costly to global medical systems as it causes unnecessary complications and expenses [5]. Accordingly, a key goal of behavioral medicine is to find ways to increase patient adherence to prescribed treatments. Therefore, from the perspective of achieving desirable clinical and economic outcomes, the influential factors that contribute to patient adherence need to be examined and better understood for developing effective strategies to promote patient adherence. An understanding of the predictive value of these factors for patient adherence would also contribute positively to the overall planning of any disease management program.

The World Health Organization Multidimensional Adherence Model identifies five interrelated dimensions of patient medication adherence: (1) social and economic factors (eg, limited access to health care facilities), (2) health care system factors (eg, provider-patient relationship and providers’ communication skills), (3) medical condition–related factors (eg, severity of symptoms), (4) therapy-related factors (eg, duration of a therapy), and (5) patient-related factors (eg, patient age, gender, and knowledge of a disease) [18,19]. Among the factors in the World Health Organization Multidimensional Adherence Model framework, considerable attention would need to be given to patients’ relevant information and knowledge [19-21]. Health care providers can meet patients’ information needs by reinforcing patient education on their treatment [19]. Educating patients about their disease status and general knowledge of their medications, as suggested in patient-centered health care, can also increase patient confidence, active participation, and patient adherence behavior [20,22]. Relatedly, a recent study revealed that personalized and repeated patient education interventions have modest efficacy in increasing patient adherence to medications [23].

However, patient education is not always *the more, the better* [20,24]. An *inverted U* relationship between knowledge and adherence has been found in adolescents [25]. Adolescent patients who know little about their therapies and illness are poor at adherence, whereas patients who are adequately educated about their disease and drug regimens are good at adherence; however, patients who know the lifelong adverse consequences might show poor medication adherence [26]. A recent clinical trial study showed that patient education significantly improved medication adherence but found no differences between single- and multicomponent education interventions [20]. Given the importance of patient education for patient adherence and the complexity of this relationship, further studies are needed to understand the underlying complex relationship between patient education and adherence to improve the quality of care. This knowledge gap is more meaningful, when mobile patient education programs are increasingly delivered to patients and are accessible anytime, anywhere through their mobile devices, since with traditional PC-based patient education programs, patients are primarily passive information receivers, have limited access to their health educational materials, and face difficulties in communicating with their physicians and care provider teams.

With the rapid advances and prevalence of the latest ubiquitous computing and mobile communication technologies, mobile health (mHealth) systems have been partially or fully implemented and embedded in current health care systems to foster patient-centered care. As such, increasing research has started to explore whether mHealth technologies can help improve patients’ adherence behavior. In this study, we define mHealth technologies as medical and public health practices and services, such as health care–related reminders, advice, and information delivered through mobile devices such as mobile phones [27,28]. Several studies have reported a positive relationship between the use of mHealth technologies and the increase in patients’ adherence to medication [28-30], exercise advice [31,32], and a few other contexts such as dietary behavior [33,34]. Research has also shown that a key factor that influences patient adherence is patients’ trust in their physicians and the quality of the relationships and communication between them [35-37]. Current literature also indicates that mHealth technologies can be used to increase patients’ knowledge of medical therapies and, thus, improve patient-physician communication [28,38]. However, few studies have been conducted to systematically examine how mobile technologies for patient education can be leveraged to increase patients’ trust in physicians and further improve patients’ adherence behavior.

### Objectives

Therefore, in this study, we aimed to fill such a knowledge gap to identify and empirically examine in-depth mechanisms through a lens of theory of planned behavior (TPB) on how and why such patient-physician trust is formed and leveraged by a mobile patient education system (MPES), leading to increased actual adherence to prescribed medical therapies. Thus, we conducted a field survey of 125 patients in multiple states in the United States who have used an innovative mHealth system for their health care education, information seeking, and communication with physicians. Our main finding was that patient-physician communication and the use of an MPES significantly increased patients’ trust in their physicians, which further influenced patient attitude, intention, and actual behavior toward treatment adherence.

## Methods

### Research Model and Hypotheses

**Figure 1:**
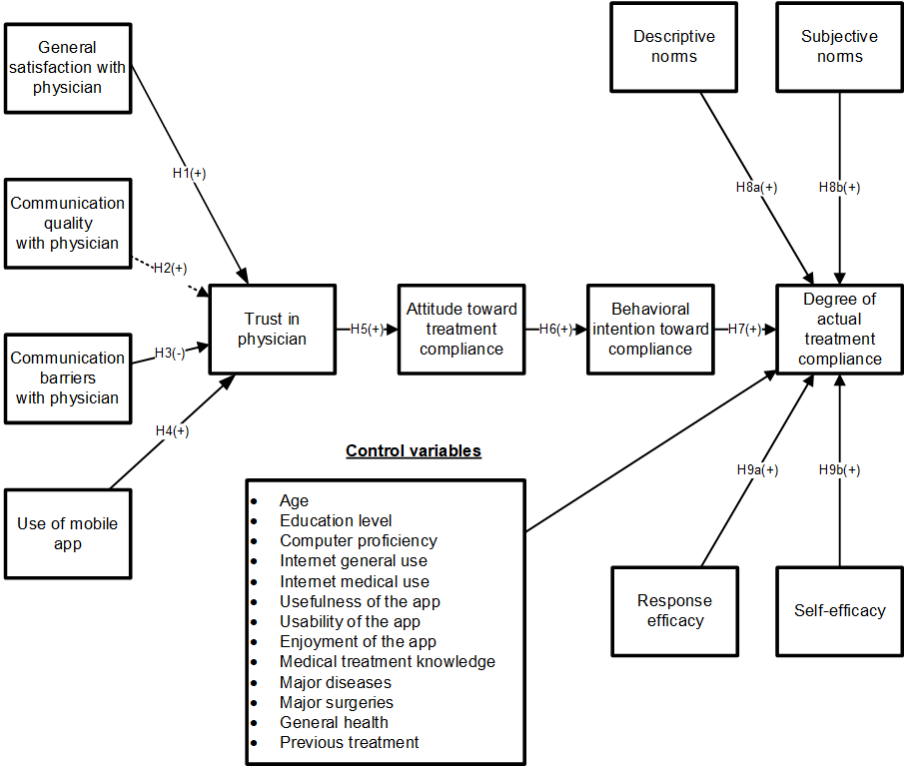
The research model. H1: hypothesis 1; H2: hypothesis 2; H3: hypothesis 3; H4: hypothesis 4; H5: hypothesis 5; H6: hypothesis 6; H7: hypothesis 7; H8a: hypothesis 8a; H8b: hypothesis 8b; H9a: hypothesis 9a; H9b: hypothesis 9b.

In this subsection, we propose our theoretical model and develop hypotheses to explain the role of an MPES in a health care setting, where an MPES affects patients’ trust in physicians, which further influences their adherence. Our model is presented in Figure 1. First, we draw on the interpersonal trust literature and hypothesize the determinants of interpersonal trust. Specifically, we explain that one’s general satisfaction and communication quality with one’s physician helps form a patient’s trust in physicians, whereas communication barriers with physicians decrease trust. Moreover, we specifically explain how the use of an MPES designed to increase patients’ understanding of treatments may also increase trust. Finally, we draw on the well-known TPB [39] to explore how a patient’s trust in physicians enhanced by an MPES may influence their treatment adherence, along with other factors that are derived from the TPB. On the basis of the existing literature, we also included some related covariates in our model to examine other factors that may affect patient adherence.

Trust has been widely studied and recognized as a cornerstone of effective patient-physician relationships [36,40-43]. In this study, we focus on interpersonal trust, which is defined as *the extent to which a person is confident in and willing to act on the basis of, the words, actions, and decisions of another* [44]. Interpersonal trust has been widely studied in the information systems literature [45,46]. In this context, we define trust as the acceptance of a vulnerable situation in which the patient believes that the physician will act in the patient’s best interests and provide assistance and support for medical care and treatment [43,47]. The vulnerable situation and the need for trust are associated with being unhealthy, the information asymmetries of medical knowledge, and the uncertainty of risks regarding the intentions and competence of the physician [40].

Next, we focus on discussing 3 key factors and their underlying mechanisms associated with patients’ trust: patients’ general satisfaction, communication quality, and communication barriers between physicians and patients. Patients’ general satisfaction reflects their perceptions and attitudes toward physicians and medical care in general [48]. Patients’ general satisfaction with care signals good relationships between them and physicians [49]. It also reflects patients’ perception of the physician’s effective treatment in previous medical care, indicating that the physician will have the ability to provide high-quality medical care and treatment in the future. Hence, patients’ general satisfaction affects their confidence and trust in the physician for medical care and treatment. Previous studies have also found that a higher level of patient satisfaction is associated with a higher level of patient-provider trust [49]. Thus, we hypothesize that an increase in patients’ general satisfaction with their physicians is associated with an increase in their trust in their physicians (hypothesis 1).

Communication also plays an essential role in the patient-physician relationship, especially in the effectiveness of this relationship [4,36]. Effective, sufficient, and 2-way conversations between patients and physicians enable patients to decrease the information asymmetries that come from the nature of medical knowledge and facilitate the collaborative decision-making process toward patients’ treatment plans [24,37,50], thus promoting patients’ confidence and trust in their physicians. Indeed, empirical support has been found for the effect of communication (eg, discussing options and being open during communication) on trust [51]. Conversely, barriers to effective and sufficient communication between patients and physicians, such as 1-way conversations and small talk during communication, would lead to poor understanding of the benefits and risks associated with their condition, therapy, and treatment and a poor understanding of the proper use of the medication, which would decrease patients’ confidence and trust in the physician. Previous studies have also confirmed this relationship. For example, some communication barriers may lead to a patient’s low levels of trust in physicians [22], such as when physicians answer few questions or when patients find it difficult to understand a physician’s language or writing. Too little time spent by physicians with patients is also found to threaten patients’ motivation to maintain their therapeutic treatment plan, which further leads to a low level of trust in physicians [52]. Thus, we hypothesize that an increase in patients’ communication quality with their physicians is associated with an increase in their trust in their physicians (hypothesis 2) and that an increase in patients’ communication barriers with their physicians is associated with a decrease in their trust in their physicians (hypothesis 3).

Our next hypothesis predicts that mobile patient education can help increase patients’ trust in their physicians. Our contextual assumption for the design of this study was that patients would use an MPES to learn about the treatment that they were seeking in a just-in-time manner in their physicians’ waiting room right before seeing a physician about the treatment. During this time, patients can learn the key terms, procedures, issues, risks, and benefits involved in a treatment and, thus, are able to communicate with a physician regarding their treatment plan with more knowledge and confidence. Furthermore, such an educational artifact allows basic questions to be answered ahead of time and, thus, enables patients to use the limited time with their physicians more effectively. Better and more effective communication enhances patients’ confidence and trust in their physicians.

To further explain and justify this prediction, we used several lines of reasoning and evidence. First, one of the biggest problems in trust formation between patients and physicians is poor communication and misunderstandings between them because of information asymmetry [22,53,54]. On the basis of the assumption that information is imperfect and obtaining information can be costly, information asymmetry concerns are *critical when one party lacks information about the quality of another party or when one party is concerned about another party’s behavioral tendencies* [55,56]. Information asymmetry is common in the health care sector, where physicians are equipped with their training and specialized knowledge but patients do not have similar training to physicians and usually have limited knowledge regarding their diagnoses and medical treatments [57]. Thus, we argue that an MPES can help patients educate themselves and reduce information asymmetry through the availability of relevant and timely medical information regarding their conditions and treatment plans. Enhanced knowledge and a shared understanding of the terms and issues involved in a treatment further lead to more effective communication and higher engagement during a visit. For example, by using an MPES in the waiting room, patients may have a better understanding of relevant medical information. With a more shared understanding of their condition and treatment, patients will have a 2-way conversation with their physicians to have their questions directly answered. Previous studies have also found that shared values would be tied to the production of trust, and patients who felt well informed had a generally high level of trust in their physicians [54,58].

Second, patients judge the competencies of physicians in a multifaceted way [58]. On the one hand, patients do not expect a physician to know everything. In contrast, patients seek information to judge the quality of the information they received from their physicians. Namely, they welcome evidence-based information as this helps them make self-determined decisions regarding their preferences. The possibility of making informed decisions based on rational criteria is perceived as a prerequisite for developing trust [58]. Patients can use the patient education material that they receive on their mobile devices to cross-validate the information they find from other sources and, thus, take an active role in the clinical decision-making process, helping them develop trust in their physicians [59].

Third, a key issue in the relationship between patients and physicians is too little time spent together, which undermines communication and trust [52]. Given the limited and costly time available during patients’ visits, time spent on ineffective communication would further leave less time for physicians to discuss treatment plans and address patients’ needs. Using an MPES before a time-constrained visit would empower patients with necessary and relevant medical information and allow such a visit to be more effective for both the patient and physician.

Fourth, we also assert that physicians who provide such an MPES with personalized patient materials and tools in their waiting rooms not only better prepare their patients to communicate with them about their treatments but also provide a positive signal of service quality and empathy that can facilitate patient-physician communication and trust formation [60]. Consequently, a trusting patient-provider relationship enabled by an MPES may further improve patient care quality.

In summary, using an MPES is predicted to reduce information asymmetries between patients and physicians, empower patients taking an active role in clinical decision-making processes, facilitate effective communication in a time-constrained visit, and send a message of care and empathy from the physicians, thus leading to a better patient-physician relationship and enhancing patients’ confidence and trust in their physicians. Therefore, we propose that the level of trust is likely to be higher when the level of patient use of a mobile patient education app is higher. As such, we posit that an increase in patients’ use of a mobile patient education app designed to increase their knowledge of a specific medical treatment will increase their trust in their physicians (hypothesis 4).

A trustful relationship between patients and health care service providers is key to patient adherence. In particular, a healthy relationship is established based on patients’ trust in physicians and empathy from physicians [37,60,61]. Changing patients’ attitudes and beliefs to improve their adherence behavior is more likely when there is an elevated level of patient trust. Trust in a physician correlates positively with patients’ perceived effectiveness of care, acceptance of new medications, and intention to follow physician instructions [37,50]. Thus, we hypothesize that an increase in patients’ trust in their physicians is associated with an increase in their positive attitudes toward treatment adherence (hypothesis 5).

We used the TPB to account for the formation of attitudes from beliefs, norms, and self-efficacy, which can then be used to predict subsequent behaviors [39]. Fundamental to the TPB is the idea that attitudes are the drivers of behavioral intentions, and behavioral intentions are the drivers of actual behaviors [39,62-64]. We leveraged and applied these concepts and predictions to the health care adherence context. In our model, an attitude toward treatment adherence is the primary driver of behavioral intention toward treatment adherence, which then drives actual treatment adherence behaviors. We assume that the TPB holds in this health care context; thus, we posit that an increase in positive attitudes toward treatment adherence is associated with an increase in behavioral intention toward treatment adherence (hypothesis 6) and that an increase in behavioral intention toward treatment adherence is associated with an increase in the degree of actual treatment adherence (hypothesis 7).

Next, we followed the TPB assumption that normative beliefs will influence actual adherence behaviors. Normative beliefs represent a person’s perceived social pressure to comply with a recommendation as informed by their valued social referents for the context [62,65]. Normative beliefs are also known as social influence, which comprises subjective and descriptive norms [66]. Following adherence literature [66-68], subjective norms in our context represent the degree to which patients believe that other key people (eg, family, friends, and coworkers) in their lives want them to comply with a treatment recommendation. Descriptive norms represent a patient’s beliefs about what is commonly done by most patients or the public in terms of adherence to a specific medical recommendation.

According to the TPB, norms affect individuals’ intentions and behaviors [39,62,69]. In a security context, Siponen [70] found that norms work because of the desire to conform to a group to which one belongs; this was also confirmed by Mishra et al [71]. Psychology researchers have long proposed that conformity to groups is attributable to norms and the pressures that norms place on individuals within a group [72-74]. Assuming that these social norms also play a role in patients’ decisions regarding medical recommendation adherence, we posit that an increase in descriptive norms toward a treatment is associated with an increase in the degree of actual treatment adherence (hypothesis 8a) and that an increase in subjective norms toward a treatment is associated with an increase in the degree of actual treatment adherence (hypothesis 8b).

Next, we followed the TPB assumption that one’s actual behaviors will be influenced by one’s efficacy. As a long-established component of the TPB, self-efficacy is highly important in the medical treatment adherence context as it covers patients’ basic self-assessment regarding their ability to effectively follow medical advice and whether they believe that a recommended treatment is efficacious [4,30]. In the TPB, efficacy is conceptualized as response efficacy and self-efficacy. On the basis of the literature on adherence [18,65-67], we define self-efficacy in our context as a patient’s judgment of their personal ability, competency, and knowledge in complying with a recommended medical treatment. Similarly, response efficacy is a patient’s judgment of the likely effectiveness and positive outcomes associated with a recommended medical treatment. We assume that these efficacy judgments play a role in patients’ adherence to medical recommendations and hypothesize that an increase in response efficacy toward a treatment is associated with an increase in the degree of actual treatment adherence (hypothesis 9a) and an increase in self-efficacy toward a treatment is associated with an increase in the degree of actual treatment adherence (hypothesis 9b).

### Ethical Considerations

The MPES on which we focused in this study was codeveloped by the first author’s research group and ABC Company (anonymized), which is a software company whose main products are health care systems aiming to address the communication and trust issues between patients and physicians to improve patient adherence behavior. The MPES has been successfully sold and deployed in many clinics and hospitals in North America, South America, and Asia. Patient users can access patient education materials in physicians’ clinics or anywhere else through different mobile devices. Its web portal interface is easy to navigate, and the educational contents are customized according to each patient’s health situation.

This study was approved by the institutional review board of the Southern Utah University (approval number: 15-052013). We worked with the ABC Company to obtain their support for conducting this research with their patients. With the assistance of the company’s attorney, we carefully followed the US Health Insurance Portability and Accountability Act's (HIPAA) Privacy, Security, and Breach Notification Rules to protect patients’ rights. Finally, after obtaining all approvals, we were able to post a web-based flyer on the ABC Company’s patient web portal to invite interested patients who had used the MPES to participate in our field study. We provided a US $10 honorarium for each survey respondent who provided valid and complete responses.

### Data and Sample

We conducted a field study with real patients from multiple states in the United States to test our research model. These patients were able to use an MPES designed to improve patient education and experience at their physicians’ clinics or hospitals. Patient participation was completely voluntary. They were given web-based instructions to fill out a web-based questionnaire distributed solely inside the MPES, where only active patient users could see our project flyer and answer our questions on their perceptions and assessments of using the MPES and its influence on their adherence behavior. During a period of 2 and a half months, after both plastic surgery and obstetrics versions of the MPES were launched, we received a total of 126 patient responses. We excluded 0.8% (1/126) of responses from a male patient from further data analysis because most questions were unanswered, resulting in 125 valid responses, all of which were not surprisingly from female patients, adequately representing the patient population that we reached out to. After the initial data collection, several duplicate responses were identified and removed before data analysis.

After the initial development of the questionnaire, we made it accessible on the web. We then circulated it among 26 senior students at a US university to obtain feedback on the relevance and clarity of the survey questions and on whether the web-based questionnaire could be accessed properly through different mobile devices, such as iPads, iPhones, and other types of smartphones and tablets. As we planned to deploy the questionnaire on the web, the first author also conducted a 20-minute face-to-face meeting with each student to verify the clarity of the web-based questionnaire instructions in that no face-to-face contact was expected to take place between the researchers and the actual patient respondents. According to the feedback that we obtained from these pilot sessions, we were able to further refine a few ambiguous questions and adjust the web-based questionnaire interface to better fit heterogeneous mobile platforms.

Of the 125 valid survey respondents, 110 (88%) patients were from the plastic surgery field, 9 (7.2%) were from obstetrics, and 6 (4.8%) were from other medical fields. All the respondents (125/125, 100%) were female. The average age was 39.6 (SD 12.9) years.

### Measures

For the study constructs and measures, we adapted existing validated psychometric scales and measurement items from established research. We then tailored the questions to fit the context of this study. Multimedia Appendix 1 details the key constructs and associated detailed questions found in the questionnaire. Multimedia Appendix 2 details the factor loadings as well as the means and SDs of each factor.

Items for a patient’s general satisfaction with physicians were taken from the *overall satisfaction* dimension of the patient satisfaction questionnaire scale for a specific physician designed by Ware Jr et al [48]. Items for communication barriers and constructs regarding quality of communication with physicians were adapted and modified from the scale developed by Steine et al [75]. Items related to patient trust in physicians were adopted from the study by Hall et al [76]. Use of the app was customized to the medical context based on a self-reported internet use measure [77]. Subjective and descriptive norm constructs were modified based on validated instruments originally developed by Herath and Rao [66]. The response efficacy and self-efficacy constructs were adapted from the scale developed by Workman et al [78]. Attitude toward treatment adherence, intention toward treatment adherence, and degree of actual adherence constructs were measured based on modifications of similar measures from Bulgurcu et al [65] and Hu et al [79].

### Statistical Analysis

As all our constructs were reflective and our research model contained both first-order and second-order constructs, partial least squares path modeling was used to examine our research model [80]. Partial least squares has been suggested for testing novel propositions with limited previous theoretical development [81-83], which is the nature of this study. Namely, we used SmartPLS software (version 3.0; SmartPLS GmbH) [84] to examine our research model.

In this study, we used the marker variable technique suggested by Lindell and Whitney [85] and Malhotra et al [86] to examine possible common method bias as self-reported survey data may inflate variable correlations. We selected a marker variable, organizational procedural justice [87], which is theoretically irrelevant to the context of this study. After computing the average correlation between the marker variable *organizational procedural justice* and the 12 principal constructs, which was 0.17 (average *P* value=.20), we confirmed that common method bias was not a major concern in this study.

## Results

### Descriptive Analysis

Table 1 summarizes the descriptive statistics related to the demographic information of the participants.

**Table 1:** Demographic information of the participants (N=125).

Variable and category			Participants, n (%)
**Sex**			
	Female	125 (100)	
	Male	0 (0)	
**Age (years)**			
	<20	2 (1.6)	
	20 to 29	30 (24)	
	30 to 39	33 (26.4)	
	40 to 49	35 (28)	
	50 to 59	14 (11.2)	
	≥60	11 (8.8)	
**Education level**			
	Lower than high school or secondary school	0 (0)	
	High school or secondary school	21 (16.8)	
	Some university but had not completed a degree	33 (26.4)	
	Associate degree	16 (12.8)	
	Bachelor’s degree	38 (30.4)	
	Master’s degree	13 (10.4)	
	Doctorate or PhD	4 (3.2)	
**Computer proficiency**			
	Poor	0 (0)	
	Fair	7 (5.6)	
	Good	28 (22.4)	
	Very good	53 (42.4)	
	Excellent	37 (29.6)	
**Internet general use: how often do you use the internet?**			
	Never	0 (0)	
	Seldom	0 (0)	
	Occasionally	7 (5.6)	
	Frequently	14 (11.2)	
	Always or every day	104 (83.2)	
**Internet medical use: how often do you use internet sites such as WebMD to learn about medical information?**			
	Never	2 (1.6)	
	Seldom	18 (14.4)	
	Occasionally	54 (43.2)	
	Frequently	46 (36.8)	
	Always or every day	5 (4)	

### Measurement Model

We first tested internal consistency and then examined convergent and discriminant validity of the measurement items. Composite reliability and average variance extracted (AVE) [88] were computed. Both composite reliability and AVE reached a satisfactory level (Table S1 in Multimedia Appendix 2), as suggested by Fornell and Larcker [88] that composite reliability should be >0.70 and AVE should be >0.50. As such, the internal consistency, reliabilities, and convergent validity were confirmed.

Furthermore, to verify discriminant validity, we also computed the square root of the AVE for all latent variables and compared them against their correlations with other constructs [88]. Table S1 in Multimedia Appendix 2 demonstrates that all the square roots of the AVE values are greater than their correlations with any other constructs; thus, discriminant validity was also confirmed [89].

As all factor measures loaded highly (*P*>.50) on their associated latent constructs [90], we were able to confirm both the convergent and discriminant validity of this study. As Table S2 in Multimedia Appendix 2 shows, all items are >0.70 on their targeted constructs, which are much higher than the suggested threshold (*P*>.50) and other cross-loadings. Therefore, the results support convergent and discriminant validity [91].

### Structural Model

**Figure 2:**
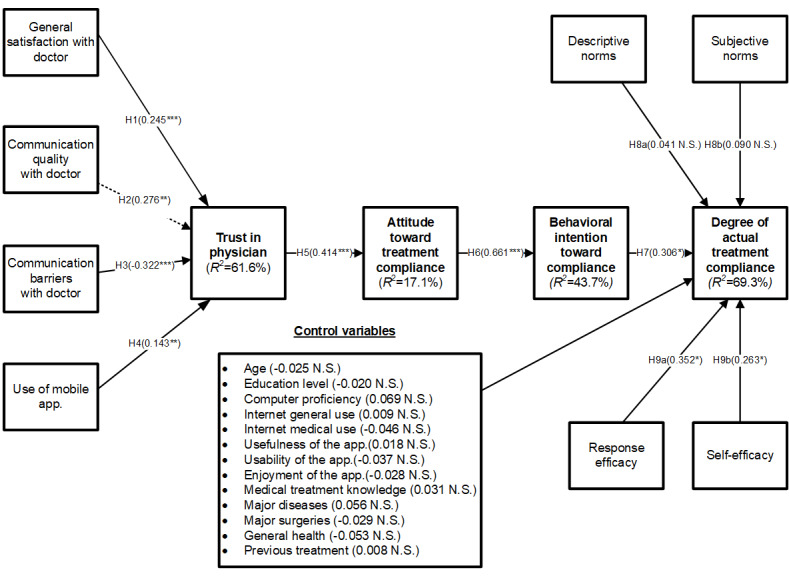
Results of research model testing. *P<.05, **P<.01, ***P<.001; H1: hypothesis 1; H2: hypothesis 2; H3: hypothesis 3; H4: hypothesis 4; H5: hypothesis 5; H6: hypothesis 6; H7: hypothesis 7; H8a: hypothesis 8a; H8b: hypothesis 8b; H9a: hypothesis 9a; H9b: hypothesis 9b; N.S.: nonsignificant.

In this study, we selected and examined several control variables related to our dependent variable, *degree of actual treatment adherence*, which were age, education level, computer proficiency, internet general use, internet medical use, usefulness of the app, usability of the app, enjoyment of the app, medical treatment knowledge, major diseases, major surgeries, general health, and previous treatment. However, none of them were statistically significant, as illustrated in Figure 2. Next, we assessed the structural model to examine the path coefficients (β). We used the bootstrapping method with 500 resamples to compute the statistical significance levels of the parameter estimates. Figure 2 depicts the results. Hypotheses 1 to 4 theorized the factors that influence patient trust in physicians. General satisfaction with a physician positively affected patient trust in the physician (β=.245; *P*<.001), supporting hypothesis 1. Communication quality with a physician significantly influenced patient trust in the physician (β=.276; *P*<.01), supporting hypothesis 2. Communication barriers with a physician were negatively related to patient trust in the physician (β=−0.322; *P*<.001), supporting hypothesis 3. The use of mobile education apps was found to affect patient trust in a physician significantly and positively (β=.143; *P*<.01), supporting hypothesis 4. Furthermore, patient trust in physicians had a highly positive relationship with attitude toward treatment adherence (β=.414; *P*<.001), supporting hypothesis 5. Finally, we found that attitude toward treatment adherence was positively related to behavioral intention toward adherence (β=.661; *P*<.001), which also significantly influenced the degree of actual treatment adherence (β=.306; *P*<.05), supporting hypotheses 6 and 7, respectively. The data results also indicated that response efficacy (β=.352; *P*<.01) and self-efficacy (β=.263; *P*<.05) significantly influenced the degree of actual treatment adherence, supporting hypotheses 9a and 9b, respectively. However, subjective and descriptive norms had no influence on patients’ actual treatment adherence behavior. Thus, hypotheses 8a and 8b were not supported.

## Discussion

### Principal Findings

In this paper, we aimed to systemically examine how and why patient-physician trust is formed and how patient education delivered via an MPES influences patients’ trust and patient adherence. We leveraged the TPB along with fundamental concepts of trust to propose a model that explains how an MPES, along with other factors, can help foster trust of patients in physicians and eventually increase treatment adherence. We first confirmed that general satisfaction with physicians, communication quality, and use of an MPES jointly facilitate and foster patients’ trust in physicians, whereas communication barriers decrease trust. We also found that patients’ trust in their physicians was indeed a significant determinant of positive attitude formation. We found compelling evidence for our expanded model based on the TPB. Attitudes toward treatment adherence were positively related to intentions toward treatment adherence, and these intentions were positively related to actual treatment adherence behaviors. In addition to behavioral intention toward adherence, we found that response efficacy and self-efficacy enhance actual treatment adherence behaviors. A key exception in our model was the insignificant influence of social norms (ie, subjective and descriptive norms). This was particularly surprising in the context of plastic surgery visits, in which we expected social norms to play a stronger role. As most of our study participants were patients of plastic surgery who primarily sought treatment to improve their physical appearance instead of for medical reasons, the insignificant results associated with social norms were likely caused by their privacy concerns, which requires further research. Our research model explained 61.9% of the variance (*R*^2^) in patient trust in physicians, 17.1% of the attitude toward treatment adherence, 43.7% of the behavioral intention toward adherence, and 69.3% of the variance of patients’ actual adherence behavior.

### Comparison With Prior Work

First, as one of the first studies that systematically examine the relationship between the use of mobile technologies and patients’ trust in physicians in the context of patients being active users of technologies, we found that patient use of an MPES positively influences patient trust in physicians. Previous studies have examined patients’ trust in technologies used solely by providers, such as electronic health records, electronic monitors, and web-based health communities supported by the internet [92-94]. In most previous studies, patients had limited control over the technologies and were passive users of them [92]. In our study, patients were active users of technologies and had control over how and when to use and access the content on mobile devices. In current patient-centered health care, the role of patients has evolved to becoming those active partners; thus, it is crucial to consider the influence of patients’ use of health care technology in this relationship when mobile technologies become more prevalent ways of conducting patient outreach and intervention programs. Our study enriches the trust literature by addressing the call to identify key determinants of the use of patient-centered mobile technologies on trust.

Second, our study proposed an integrated model to explain how communication, patient satisfaction, MPES use, and trust foster the degree of actual treatment adherence. We examined these 4 factors in the patient adherence context. We found that communication, patient satisfaction, and MPES use jointly influence trust, which further fosters actual patient adherence. Overall, the integration of factors from multiple streams of the literature to explain patient adherence behavior is one of our core theoretical contributions, especially from a TPB theoretical lens. This finding advances our understanding of underlying patient adherence mechanisms.

Third, we examined an MPES in a clinical setting. Although the focus of our study, patient education mechanisms, is not new to the patient adherence literature, previous relevant patient adherence research has not examined patient education mechanisms delivered through mobile systems. Given the popularity and availability of mobile devices, patient education is increasingly delivered and communicated through mobile devices. Previous patient education studies have primarily focused on engaging patients in mHealth intervention programs to improve their adherence behaviors regarding medication [28-30], exercise advice [31,32], and dietary behavior [33,34] and have mostly studied 1-way communications between patients as passive users and their physicians. However, none of these studies explored the role of personalized patient education intervention programs in an mHealth environment to systematically understand how to establish and improve patients’ trust in physicians, which is critical to improving patient-centered care. Our study provides an in-depth understanding of the influence of patient education mechanisms delivered through an MPES on patient adherence.

Finally, we extended and empirically tested the TPB in the context of patient adherence. From a theoretical standpoint, few empirical studies have examined the TPB in the context of patient adherence [30]; thus, our study extended the TPB to an mHealth context and provided empirical evidence on how an MPES can leverage patient trust in physicians to improve patient adherence, confirming that the TPB also holds in this context.

### Strengths

It is critical for physicians to understand how to enhance patient adherence to their treatment recommendations. Our study indicates that an approach that may help is for physicians to proactively leverage mobile technologies such as an MPES to enhance the provider-patient relationship and foster patient trust in physicians. We recommend that physicians consider the trust-building process both on the web and in the office. Web-based trust can be developed by providing quality apps for patients to adopt and use while considering patients as active partners in the care process. Offline trust can be built through 2-way effective conversations with patients by addressing their personalized treatment needs. This study also highlights the importance of choosing the appropriate mobile technologies and apps for patients to use given that a message of care and empathy to patients disseminated through an MPES can further increase patients’ trust in physicians and enhance patient adherence.

Our results also imply that additional changes to clinical workflows in hospitals and clinics may enhance patient-centered care. For example, the Mayo Clinic, as the leading hospital system in the United States, has implemented a secure patient message portal system to improve communication quality between physicians and patients, patient engagement, and patient-centered care [95]. Moreover, in practice, teams of caregivers—including hospitals, clinicians, nursing practitioners, and physician assistants—may also need to find effective ways to balance their main workload to take care of patients and handle increasingly growing communication loads with their patients to achieve the desired clinical operation efficiency and higher care quality driven by improved patient adherence. Thus, our study provides in-depth mechanisms to further this area of health care practice to not only foster patient engagement and communication but also establish physician-patient trust, which is critical to patient adherence and care quality.

### Limitations

Our study has several limitations owing to the nature of the field study but also provides new opportunities for future research. First, this field study was restricted to physicians who had adopted the MPES in their practices and were willing to offer us access to their patients. Although all valid survey respondents were female, they were representative of the population in the related medical practices—plastic surgery and obstetrics.

Second, after going through many complicated legal and research coordination processes, we were only allowed to conduct the study with patients who had tried the MPES; thus, we were not able to reach patients who were still using traditional patient education systems as a control group. Patient adherence behavior has not been well studied across populations, diseases, and settings, thus making it difficult for health professionals and patients to know which strategies work and which do not [96]. We suggest that researchers further study the use of MPES in this context but involve other types of patients—including a balance of gender—such as those with chronic diabetes, mental illness, or cardiovascular disease who have serious adherence challenges in an mHealth setting.

Third, according to the behavior model of persuasive design by Fogg [97] and more personally controlled help-seeking features suggested by Lau et al [98], it would be valuable to conduct a longitudinal field study to observe patients’ adherence behaviors during their different treatment stages in comparison with a control group. Moreover, we may also explore how different types of mobile interface designs and new technologies such as radio-frequency identification and the Internet of Things affect both patients’ adherence behavior and physicians’ decision-making processes [99]. Conversely, as mHealth systems represent innovative technology, many hospitals and physicians’ offices have not adopted such systems to benefit their patients. However, how physicians can make more informed decisions to address patients’ nonadherence issues through an MPES more effectively warrants further investigation.

### Conclusions

In summary, extensive research has been conducted that examines factors associated with patient adherence, some of which has examined the relationship between patient education and adherence. However, as highlighted by extant literature, the underlying relationship between patient education and adherence is complex, and no studies to date have been conducted that explore and explain the underlying mechanisms of patient education delivered through mobile devices on patient adherence [24,100]. Achieving a more in-depth understanding of the effects of these mechanisms on adherence can have theoretical and practical implications on how to leverage an MPES to improve patient adherence, which in turn may improve health care outcomes. Thus, our study aimed to bridge this compelling knowledge gap.

In this study, our MPES provided additional patient care at the physician’s office or clinic through a real-time mobile personalized patient education intervention program, which enabled 2-way physician-patient communication beyond the patients’ in-person office visits. Our study participants were active patient users of the MPES, on which they could access their individual patient education materials and directly interact with their physicians and caregiver teams on the web. The results of our study imply that the MPES can be effectively leveraged by physicians’ offices or clinics for more seamless high-quality care. It can also be a trade-off for physicians’ offices or clinics to handle additional workload to provide more personalized services to their patients through an MPES. Nevertheless, our study findings indicate that the extended service lines provided on the MPES beyond regular in-person office visits may significantly improve patient-physician communication quality and increase patients’ trust in their physicians, thus leading to more optimal health outcomes such as enhanced patient adherence to their therapy or treatment plans.

In conclusion, our study is one of the first that examines the relationship between patients who actively use an MPES and their trust in their physicians. This study contributes to this context by (1) enriching the trust literature addressing the call to identify key patient-centered technology determinants of trust, (2) advancing the understanding of patient adherence mechanisms, (3) adding a new explanation for the influence of education mechanisms delivered through mobile devices on patient adherence, and (4) confirming that the TPB holds in this patient adherence context.

## Appendix

Multimedia Appendix 1 Patient survey instrument and questions and measures for the theoretical model.

Multimedia Appendix 2 Measurement model statistics and validity details.

## Abbreviations

AVE: average variance extracted
mHealth: mobile health
MPES: mobile patient education system
TPB: theory of planned behavior

## Data Availability

Access to the data set analyzed in this study may not be available to researchers because of Health Insurance Portability and Accountability Act and institutional review board constraints.
