# Supplementary material for: Patient Trust in Physicians Matters—Understanding the Role of a Mobile Patient Education System and Patient-Physician Communication in Improving Patient Adherence Behavior: Field Study
Source: J Med Internet Res. 2022 Dec 20;24(12):e42941. doi: 10.2196/42941 (PMC9776535; doi:10.2196/42941)
Supplement: Multimedia Appendix 1 [file jmir_v24i12e42941_app1.docx]

## Multimedia Appendix 1. Patient survey instrument and questions and measures for the theoretical model.

##

The purpose of this survey is to gain a better understanding about the benefits of using ABC patient education system offered by your physician's office. Your valuable feedback will be used to further improve our patient education system, which will benefit other patients throughout the United States. The whole survey will take you approximately 10–15 minutes.

Please answer the following questions with your TRUE opinions on the ABC system you are using. There are no wrong or right answers, and we just want your honest opinions. Accordingly, there are few risks involved with this survey other than being asked questions you might not know or may not want to answer. At no time will your privacy and confidentiality in replying to this questionnaire be violated. Individual-level results will NOT be released or analyzed, nor will they be available for your physician to review. Your names will be expunged permanently from the dataset once we verify you have completed the survey for compensation purposes. All data will be anonymized and averaged. Thank you in advance for your highly valued feedback.

Note: In exchange for your time and feedback, we will a provide US$10 honorarium/person for the first 150 online survey respondents after we confirm that their survey responses are complete and valid. If you have never used the patient education system offered by your physician 's office, you are not eligible for this survey.

Continuing with this survey indicates that you understand any potential risks and that you fully agree to participate. You may stop participation at any time. However, only those who provide valid and complete answers to the entire survey are eligible for compensation.

### Demographic and Covariate Questions

- **Gender**: What is your gender? (1 = Female, 2 = Male)
- **Age**: What is your age? ___
- **Education level**: What is your educational level?

1 = Less than high school/secondary school

2 = High school/secondary school

3 = Some university, but have not completed a degree

4 = associate degree

5 = Bachelor’s degree

6 = Master’s degree

7 = Doctorate/Ph.D.

- The name of your physician’s office (e.g., Davis Plastic Surgery Clinic) is ____
- Your physician’s area of expertise: 1) Plastic Surgery; 2) Obstetrics; 3) Dermatology; 4) Other (please specify:_________)
- **Where have you experienced the ABC system?** (1 = only at my physician’s office; 2 = only at home; 3 = only at work; 4 = only on travel; 5 = both at physician’s office and at home; 6 = at physician’s office, home, and work; 7 = all of the above; 8 = none of the above)
- **Note: If your answer to the above question—"Where have you experienced the ABC system?"—is "none of the above," please stop here. Thank you for your kind participation!**

| Organizational justice: Procedural justice; Adapted from [87] | Consider the company/organization you currently work for or previously worked for and indicate the degree to which you believe the following to be true: “My organization’s procedures…”   1. …are fair. 2. …are applied consistently. 3. …are free of bias. 4. …uphold high ethical standards.   [5-point scale: 1 = never, 2 = rarely, 3 = sometimes, 4 = most of the time, 5 = always]  [Marker variable for CMV analysis was chosen because it is an organizational-level variable and thus should be unrelated to anything in this study.] |
| --- | --- |

- **What kind of device(s) have you used to access this patient education system? (Checkbox:** 1 = iPhone; 2 = Android phone; 3 = Other smartphones; 4 = iPad; 5 = Kindle; 6 = Other tablets; 7 = Regular PC; 8 = Big touchscreen monitor PC; 9 = Not applicable)
- **Computer proficiency:**

How would you evaluate your computer skills in general? [1 = poor, 2 = fair, 3 = good, 4 = very good, 5 = excellent]

- **Internet general use:** How often do you use the Internet?

[1 = never, 2 = seldom, 3 = occasionally, 4 = frequently, 5 = always/every day]

- **Internet medical use:** How often do you use Internet sites such as WebMD to learn about medical information?

[1 = never, 2 = seldom, 3 = occasionally, 4 = frequently, 5 = always/every day]

- **Please share your experiences with the ABC system so far:**
- **Usefulness of application:** To what degree would you agree that the ABC application is useful to you?

[1 = strongly disagree, 2 = disagree, 3 = somewhat disagree, 4 = neither agree/disagree, 5 = somewhat agree, 6 = agree, 7 = strongly agree]

- **Usability of application:** To what degree would you agree that the ABC application is easy to use?

[1 = strongly disagree, 2 = disagree, 3 = somewhat disagree, 4 = neither agree/disagree, 5 = somewhat agree, 6 = agree, 7 = strongly agree]

- **Enjoyment of application:** To what degree would you agree that the ABC application is enjoyable to use?

[1 = strongly disagree, 2 = disagree, 3 = somewhat disagree, 4 = neither agree/disagree, 5 = somewhat agree, 6 = agree, 7 = strongly agree]

- **Medical treatment knowledge:** Please rate the degree to which you believe you are knowledgeable about the medical treatment that you’re pursuing.

[1 = no knowledge at all, 2 = not very knowledgeable, 3 = somewhat knowledgeable, 4 = knowledgeable, 5 = highly knowledgeable]

- **Major diseases:** Have you suffered from or are you currently suffering from major diseases or medical conditions that threaten your general health and life? (e.g., cancer, cardiovascular disease, obesity, diabetes, strokes, etc.) [1 = yes/2 = no]
- **Major surgeries?** Have you ever had a major surgery that required general anesthesia and at least one night’s stay in a hospital? [1 = yes/2 = no]
- **General health:** Please rate your general health:

[1 = extremely poor health, 2 = poor health, 3 = neither bad/good health, 4 = good health, 5 = extremely good health]

- **# Prior treatments:** Counting this visit, approximately how many total times have you visited this physician for this particular course of treatment or medical concern? ____
- **Treatment sought:** Please describe the general kind of treatment that you’re seeking with this physician’s office that uses the ABC system. ____

## Part II: Questions and Measures for Theoretical Model

Table S.1. Model Constructs and Measures

| **Construct** | **Item** | **Item Wording** | **Factor Loading** | **Mean (SD)** |
| --- | --- | --- | --- | --- |
| General satisfaction with physician (GSP)  Taken from the “overall satisfaction” dimension of the patient satisfaction questionnaire (PSQ) scale, for specific doctor [48] | GSP1 | I am very satisfied with the medical care I have received from this physician. | 0.70 | 6.23 (1.22) |
|  | GSP2 | The care I have received from this physician is just about perfect. | 0.88 | 6.01 (1.01) |
|  | GSP3(r) | There are things about the medical care I receive from this physician that could be better. | 0.76 | 5.18 (1.78) |
| Communication quality with physician (CQP)  [75] | CQP1 | We have good conversations. | 0.91 | 6.05 (0.93) |
|  | CQP2 | This physician understands my needs. | 0.92 | 6.22 (0.83) |
|  | CQP3 | This physician understands what is in my mind. | 0.90 | 5.80 (1.23) |
|  | CQP4 | When talking to this physician, I feel I am being taken care of and attended to. | 0.83 | 6.13 (1.13) |
| Communication barriers with physician (CBP)  [75] | CBP1 | We spend too much time on small talk. | 0.70 | 1.89 (0.99) |
|  | CBP2 | It is difficult for me to ask questions. | 0.88 | 1.97 (1.31) |
|  | CBP3 | I don’t understand important decisions being made by my physician. | 0.77 | 1.77 (1.04) |
|  | CBP5 | It is a bit difficult to connect with this physician. | 0.85 | 1.77 (1.12) |
| Use of mobile app. (UA); modified from Horton, Buck [77] | UA1 | To what degree would you say that you have used the ABC app. to prepare for your physician visits and to learn more about treatment options?  *(Scale: 1 = never, 2 = seldom, 3 = occasionally, 4 = frequently, 5 = always or every visit)* | 0.89 | 2.75 (1.18) |
|  | UA2 | Approximately how many times in total have you used ABC app. offered by your physician's office?  *(Scale: 1 = one or two times; 2 = three or four times; 3 = five or six times; 4 = more than six times)* | 0.78 | 1.81 (1.05) |
| Trust in physician (T)  [76] | T1 (r) | Sometimes this physician cares more about what is convenient for him or her than about your medical needs. | 0.74 | 5.83 (1.34) |
|  | T2 | This physician is extremely thorough and careful. | 0.72 | 6.03 (1.43) |
|  | T3 | You completely trust this physician’s decisions about which medical treatments are best for you. | 0.80 | 6.21 (0.98) |
|  | T4 | This physician is totally honest in telling you about all the different treatment options available for your condition. | 0.80 | 6.14 (1.18) |
|  | T5 (r) | Sometimes this physician does not pay full attention to what you are trying to tell him or her. | 0.85 | 5.96 (1.33) |
|  | T6 | You have no worries about putting your life in this physician’s hands. | 0.82 | 5.92 (1.34) |
|  | T7 | All in all, you have complete trust in this physician. | 0.88 | 6.13 (1.18) |
| Subjective norms (SN)  Modified for medical context from Herath and Rao [66] | SN1 | My family members think I should comply with my physician’s medical treatment. | 0.90 | 5.99 (1.22) |
|  | SN2 | My friends think I should follow my physician’s recommended course of treatment. | 0.85 | 5.86 (1.26) |
|  | SN3 | My colleagues at work or school think I should follow my physician’s orders. | 0.84 | 5.53 (1.35) |
| Descriptive norms (DN)  Modified for medical context from Herath and Rao [66] | DN1 | I believe other patients follow their physician’s recommendations. | 0.79 | 5.38 (1.45) |
|  | DN2 | I am convinced that other patients comply with their physician s’ suggested course of treatment. | 0.93 | 5.24 (1.23) |
|  | DN3 | It is likely that the majority of other patients follow their physician s’ orders. | 0.81 | 5.31 (1.17) |
| Response efficacy (RE)  Modified for medical context from Workman, Bommer [78] | RE1 | The available choices that I can take to comply or follow with my physician’s recommended course of treatment are effective. | 0.90 | 6.16 (0.84) |
|  | RE2 | The methods, information, and tools available to me help me comply with or follow my physician’s orders. | 0.93 | 6.18 (0.83) |
|  | RE3 | If I perform the suggested measures available to me, I can effectively comply with or follow my physician’s treatment plan. | 0.92 | 6.30 (0.75) |
| Self-efficacy (SE)  Modified for medical context from Workman, Bommer [78] | SE1 | For me, taking the actions needed to comply with or follow my physician’s recommended treatment is easy. | 0.78 | 5.82 (1.06) |
|  | SE2 | I have the necessary skills and knowledge to comply with or follow my physician’s orders. | 0.89 | 6.39 (0.73) |
|  | SE3 | My skills and knowledge required to adhere to my physician’s suggested medical treatment are adequate. | 0.89 | 6.34 (0.75) |
| Attitude toward adherence (ATA)  Modified for medical context from Bulgurcu, Cavusoglu [65] | To me, complying with/following the requirements of my physician’s recommended treatment is … | | | |
|  | ATC1 | …necessary | 0.90 | 6.22 (1.13) |
|  | ATC2 | …beneficial | 0.91 | 6.46 (0.78) |
|  | ATC3 | …important | 0.93 | 6.42 (0.85) |
| Intention toward treatment Adherence (ITTA)  Modified for medical context from Bulgurcu, Cavusoglu [65] and Hu, Xu [79] | To what degree do you intend to comply with or follow or NOT comply with or NOT follow the requirements of this physician’s recommended course of treatment for you? | | | |
|  | ITTC1 | I intend to comply or follow with the requirements. | 0.97 | 6.57 (0.63) |
|  | ITTC2 | I intend to adhere to the prescribed course of treatment. | 0.81 | 6.46 (0.90) |
|  | ITTC3 | I intend to carry out my responsibilities prescribed in the requirements. | 0.94 | 6.56 (0.64) |
| Degree of actual treatment adherence (DATA)  Modified from behavioral intent for medical context from Bulgurcu, Cavusoglu [65] and Hu, Xu [79] | Considering your current behavior, how strongly do you follow this physician’s recommended course of action each day? | | | |
|  | DATA1 | I comply with or follow the requirements fully. | 0.98 | 6.29 (0.81) |
|  | DATA2 | I fully carry out my responsibilities prescribed in the requirements. | 0.98 | 6.27 (0.79) |

Note: All constructs except “Use of the app” were measured on a Likert-type 1–7 scale, as follows: 1 = strongly disagree, 2 = disagree, 3 = somewhat disagree, 4 = neither agree or disagree, 5 = somewhat agree, 6 = agree, 7 = strongly agree.
