# Supplementary material for: Patient Trust in Physicians Matters—Understanding the Role of a Mobile Patient Education System and Patient-Physician Communication in Improving Patient Adherence Behavior: Field Study
Source: J Med Internet Res. 2022 Dec 20;24(12):e42941. doi: 10.2196/42941 (PMC9776535; doi:10.2196/42941)
Supplement: Multimedia Appendix 2 [file jmir_v24i12e42941_app2.docx]

## Multimedia Appendix 2. Measurement model statistics and validity details

Table S1. Internal Consistency, and Discriminant Validity of Constructs

| **Latent Construct** | **Mean** | **SD** | **AVE** | **CR** | **(1)** | **(2)** | **(3)** | **(4)** | **(5)** | **(6)** | **(7)** | **(8)** | **(9)** | **(10)** | **(11)** | **(12)** |
| --- | --- | --- | --- | --- | --- | --- | --- | --- | --- | --- | --- | --- | --- | --- | --- | --- |
| General satisfaction with physician (1) | 5.81 | 1.05 | .62 | .83 | .78 |  |  |  |  |  |  |  |  |  |  |  |
| Communication barriers with physician (2) | 1.85 | 0.88 | .64 | .88 | -.60 | .80 |  |  |  |  |  |  |  |  |  |  |
| Communication quality with physician (3) | 6.05 | 0.86 | .80 | .94 | .74 | -.71 | .89 |  |  |  |  |  |  |  |  |  |
| Use of mobile app. (4) | 2.28 | 0.94 | .70 | .82 | .19 | -.12 | .14 | .84 |  |  |  |  |  |  |  |  |
| Trust in physician (5) | 6.03 | 1.00 | .64 | .93 | .67 | -.68 | .70 | .27 | .80 |  |  |  |  |  |  |  |
| Subjective norms (6) | 5.79 | 1.10 | .75 | .90 | .19 | -.24 | .16 | .05 | .30 | .86 |  |  |  |  |  |  |
| Descriptive norms (7) | 5.31 | 1.09 | .71 | .88 | -.08 | .17 | -.18 | .06 | .01 | .19 | .85 |  |  |  |  |  |
| Response efficacy (8) | 6.21 | 0.74 | .84 | .94 | .23 | -.28 | .29 | .15 | .34 | .26 | .14 | .91 |  |  |  |  |
| Self-efficacy (9) | 6.18 | 0.72 | .73 | .89 | .26 | -.38 | .28 | .20 | .37 | .33 | .10 | .75 | .85 |  |  |  |
| Attitude toward treatment adherence (10) | 6.37 | 0.84 | .84 | .94 | .26 | -.39 | .29 | .11 | .41 | .52 | .21 | .59 | .53 | .92 |  |  |
| Intention toward adherence (11) | 6.53 | 0.65 | .83 | .93 | .27 | -.39 | .27 | .10 | .38 | .41 | .18 | .51 | .54 | .66 | .91 |  |
| Degree of actual adherence (12) | 6.28 | 0.79 | .96 | .98 | .19 | -.24 | .20 | .15 | .30 | .39 | .20 | .72 | .71 | .62 | .67 | .98 |

Note: CR: Composite Reliability; SD = standard deviation; diagonal elements highlighted in grey: the square root of the AVE of latent constructs. Off-diagonal elements: the correlations between latent constructs.

Table S2. Loading and Cross-Loading of Measures

| **Constructs** | **Item** | **(1)** | **(2)** | **(3)** | **(4)** | **(5)** | **(6)** | **(7)** | **(8)** | **(9)** | **(10)** | **(11)** | **(12)** |
| --- | --- | --- | --- | --- | --- | --- | --- | --- | --- | --- | --- | --- | --- |
| General satisfaction with physician (1) | GSP1 | .70 | -.38 | .46 | .14 | .39 | .08 | -.12 | .13 | .14 | .15 | .19 | .06 |
|  | GSP2 | .88 | -.54 | .72 | .17 | .62 | .19 | -.04 | .24 | .26 | .27 | .31 | .24 |
|  | GSP3 | .76 | -.47 | .52 | .14 | .53 | .14 | -.06 | .15 | .19 | .18 | .12 | .11 |
| Communication barriers with physician (2) | CBP1 | -.41 | .70 | -.54 | -.08 | -.46 | -.11 | .15 | -.27 | -.23 | -.33 | -.16 | -.08 |
|  | CBP2 | -.48 | .88 | -.57 | -.14 | -.58 | -.19 | .14 | -.26 | -.38 | -.31 | -.38 | -.25 |
|  | CBP3 | -.39 | .77 | -.44 | -.08 | -.45 | -.28 | .12 | -.19 | -.34 | -.28 | -.43 | -.19 |
|  | CBP4 | -.60 | .85 | -.68 | -.08 | -.65 | -.19 | .13 | -.19 | -.28 | -.34 | -.28 | -.23 |
| Communication quality with physician (3) | CQP1 | .63 | -.62 | .91 | .14 | .60 | .15 | -.21 | .19 | .22 | .20 | .21 | .12 |
|  | CQP2 | .72 | -.66 | .92 | .17 | .67 | .11 | -.16 | .27 | .29 | .26 | .21 | .20 |
|  | CQP3 | .67 | -.71 | .90 | .15 | .67 | .20 | -.11 | .32 | .28 | .40 | .32 | .20 |
|  | CQP4 | .60 | -.63 | .83 | .05 | .55 | .12 | -.16 | .24 | .20 | .16 | .22 | .18 |
| Use of mobile app. (4) | UA1 | .15 | -.12 | .11 | .89 | .26 | .06 | .07 | .15 | .22 | .14 | .08 | .12 |
|  | UA2 | .18 | -.08 | .13 | .78 | .18 | .02 | .02 | .10 | .10 | .03 | .10 | .13 |
| Trust in physician (5) | T1 | .49 | -.45 | .47 | .27 | .88 | .26 | .09 | .26 | .33 | .32 | .36 | .30 |
|  | T2 | .64 | -.67 | .70 | .16 | .74 | .21 | -.18 | .26 | .23 | .37 | .25 | .22 |
|  | T3 | .47 | -.44 | .48 | .20 | .72 | .24 | .07 | .13 | .22 | .25 | .22 | .21 |
|  | T4 | .64 | -.61 | .67 | .14 | .80 | .26 | -.02 | .37 | .39 | .36 | .39 | .34 |
|  | T5 | .45 | -.50 | .45 | .29 | .80 | .18 | .06 | .28 | .25 | .22 | .26 | .21 |
|  | T6 | .54 | -.64 | .61 | .21 | .85 | .24 | .01 | .26 | .26 | .35 | .29 | .18 |
|  | T7 | .44 | -.42 | .47 | .27 | .82 | .29 | .07 | .29 | .37 | .41 | .36 | .25 |
| Subjective norms (6) | SN1 | .19 | -.23 | .18 | .05 | .28 | .90 | .15 | .27 | .35 | .51 | .43 | .43 |
|  | SN2 | .17 | -.23 | .16 | .04 | .30 | .85 | .15 | .18 | .17 | .40 | .34 | .24 |
|  | SN3 | .12 | -.15 | .07 | .02 | .21 | .84 | .21 | .21 | .29 | .41 | .27 | .31 |
| Descriptive norms (7) | DN1 | -.09 | .15 | -.12 | .12 | .11 | .19 | .79 | .05 | .07 | .15 | .12 | .09 |
|  | DN2 | -.07 | .14 | -.11 | .07 | .02 | .18 | .93 | .16 | .14 | .17 | .16 | .22 |
|  | DN3 | -.06 | .15 | -.22 | -.01 | -.08 | .14 | .81 | .09 | .03 | .21 | .16 | .15 |
| Response efficacy (8) | RE1 | .21 | -.32 | .26 | .16 | .32 | .23 | .15 | .90 | .62 | .53 | .45 | .59 |
|  | RE2 | .22 | -.28 | .27 | .10 | .33 | .28 | .11 | .93 | .66 | .57 | .49 | .63 |
|  | RE3 | .19 | -.19 | .25 | .15 | .28 | .21 | .13 | .92 | .75 | .53 | .47 | .74 |
| Self-efficacy (9) | SE1 | .18 | -.23 | .15 | .11 | .23 | .17 | .16 | .60 | .78 | .36 | .33 | .52 |
|  | SE2 | .26 | -.43 | .26 | .15 | .39 | .32 | .04 | .68 | .85 | .51 | .53 | .58 |
|  | SE3 | .22 | -.31 | .29 | .23 | .32 | .34 | .08 | .64 | .84 | .48 | .50 | .69 |
| Attitude toward treatment adherence (10) | ATA1 | .21 | -.33 | .20 | .02 | .33 | .47 | .12 | .47 | .43 | .90 | .54 | .56 |
|  | ATA2 | .32 | -.43 | .35 | .14 | .45 | .41 | .17 | .55 | .50 | .91 | .62 | .49 |
|  | ATA3 | .18 | -.32 | .24 | .13 | .35 | .54 | .27 | .60 | .51 | .93 | .65 | .66 |
| Intention toward adherence (11) | ITTA1 | .27 | -.38 | .27 | .13 | .39 | .41 | .19 | .51 | .54 | .66 | .97 | .67 |
|  | ITTA2 | .18 | -.28 | .21 | .00 | .23 | .30 | .07 | .33 | .36 | .47 | .81 | .45 |
|  | ITTA3 | .28 | -.38 | .27 | .12 | .40 | .40 | .20 | .53 | .54 | .65 | .94 | .66 |
| Degree of actual adherence (12) | DATA1 | .18 | -.25 | .19 | .14 | .30 | .40 | .19 | .67 | .68 | .62 | .65 | .98 |
|  | DATA2 | .19 | -.22 | .20 | .15 | .29 | .37 | .20 | .74 | .70 | .60 | .65 | .98 |
